# Effects of 20 Selected Fruits on Ethanol Metabolism: Potential Health Benefits and Harmful Impacts

**DOI:** 10.3390/ijerph13040399

**Published:** 2016-04-01

**Authors:** Yu-Jie Zhang, Fang Wang, Yue Zhou, Ya Li, Tong Zhou, Jie Zheng, Jiao-Jiao Zhang, Sha Li, Dong-Ping Xu, Hua-Bin Li

**Affiliations:** 1 Guangdong Provincial Key Laboratory of Food, Nutrition and Health, Department of Nutrition, School of Public Health, Sun Yat-Sen University, Guangzhou 510080, Guangdong Province, China; zhyujie3@mail2.sysu.edu.cn (Y.-J.Z.); missingfeng@yeah.net (F.W.); zhouyue3@mail2.sysu.edu.cn (Y.Z.); saferide@126.com (Y.L.); zt740359815@163.com (T.Z.); ziyuzile_0215@163.com (J.Z.); zhangjj46@mail2.sysu.edu.cn (J.-J.Z.) dongpxu@163.com (D.-P.X.); 2 School of Chinese Medicine, The University of Hong Kong, Hong Kong, China; lishasl0308@163.com; 3 South China Sea Bioresource Exploitation and Utilization Collaborative Innovation Center, Sun Yat-Sen University, Guangzhou 510006, Guangdong Province, China

**Keywords:** alcohol, metabolism, fruit, hepatoprotection, harmful impact

## Abstract

The consumption of alcohol is often accompanied by other foods, such as fruits and vegetables. This study is aimed to investigate the effects of 20 selected fruits on ethanol metabolism to find out their potential health benefits and harmful impacts. The effects of the fruits on ethanol metabolism were characterized by the concentrations of ethanol and acetaldehyde in blood, as well as activities of alcohol dehydrogenase and acetaldehyde dehydrogenase in liver of mice. Furthermore, potential health benefits and harmful impacts of the fruits were evaluated by biochemical parameters including aspartate transaminase (AST), alanine transferase (ALT), malondialdehyde, and superoxide dismutase. Generally, effects of these fruits on ethanol metabolism were very different. Some fruits (such as *Citrus limon* (yellow), *Averrhoa carambola*, *Pyrus* spp., and *Syzygium samarangense*) could decrease the concentration of ethanol in blood. In addition, several fruits (such as *Cucumis melo*) showed hepatoprotective effects by significantly decreasing AST or ALT level in blood, while some fruits (such as *Averrhoa carambola*) showed adverse effects. The results suggested that the consumption of alcohol should not be accompanied by some fruits, and several fruits could be developed as functional foods for the prevention and treatment of hangover and alcohol use disorder.

## 1. Introduction

Alcohol consumption has been commonplace from prehistoric times. The effects of acute alcohol consumption on the human body can take several forms including facial flushing, nausea, tachycardia, palpitation, confused consciousness, and vomiting, and long-term excessive alcohol use leads to gastrointestinal damages, alcohol liver disease, pancreatitis, neurologic disorders, osteoporosis and so on [1,2,3]. The liver is the most adversely affected organ. Alcoholic liver disease encompasses a broad spectrum of progressive pathologic stages, which includes fatty liver, steatohepatitis, fibrosis, and cirrhosis [4]. Enzyme catalyzed oxidative processes exclusively metabolized most of ethanol in cytosol and mitochondria. In fact, about 90% of ethanol metabolism occurs in liver, that is, alcohol dehydrogenase (ADH) metabolizes ethanol to acetaldehyde, and then acetaldehyde dehydrogenase (ALDH) metabolizes acetaldehyde to acetate [5]. Acetaldehyde could cause toxic effects including lightheadedness, a rapid pulse, sweating, nausea, and vomiting at high concentrations. Thus, it is important to remove excess ethanol and acetaldehyde in order to prevent such toxicities [6].

Ethanol consumption results in the depletion of the endogenous antioxidants in liver. Both an increase in pro-oxidant production and a decrease in antioxidant defense most likely mediated oxidative stress [7]. Oxidative stress associated with alcohol toxicity is mainly caused by reactive oxygen species (ROS) and free radicals resulting from ethanol metabolism in liver, which initiate the peroxidation of polyunsaturated fatty acid side chains of membrane phospholipids and lipoproteins. Antioxidants could scavenge ROS and act in protecting cells from the different species of oxidants [8].

Fruits are an important nutritional resource for mankind, and contain many natural antioxidants [9,10]. The consumption of alcohol is often accompanied by other foods, including fruits. Several fruits have been reported to have an inhibitory effect on ethanol absorption and have hepatoprotective effects, while some fruits were reported to have an adverse effect after alcohol consumption [11,12]. Thus, the study on effects of fruits on ethanol metabolism is very interesting and important. In this paper, the effects of 20 selected fruits on ethanol metabolism were investigated to find out their potential health benefits and harmful impacts, and to supply new information for nutritionists and the general public to reduce harm of alcohol consumption.

## 2. Materials and Methods

### 2.1. Chemicals and Reagents

β-Nicotinamide adenine dinucleotide (NAD) and 4-methylpyrazole were purchased from Sigma Chemical Co. (St. Louis, MI, USA). Alanine aminotransferase (ALT), aspartate amino transferase (AST), superoxide dismutase (SOD), and malondialdehyde (MDA) kits were purchased from Nanjing Jiancheng Bioengineering Institute (Nanjing, China). Other chemicals were of analytical grade.

### 2.2. Fruit Materials

Twenty kinds of fruits were selected, which represented the main categories of the fruits consumed in south China. These fruits are *Actinidia chinensis*, *Averrhoa carambola*, *Chaenomeles sinensis*, *Citrullus lanatus*, *Citrus limon* (green), *Citrus limon* (yellow), *Citrus sinensis*, *Cucumis melo*, *Durio zibethinus*, *Garcinia mangostana*, *Hylocereus undulates*, *Lycopersivon esculentum*, *Mangifera indica*, *Musa nana*, *Passionfora edulis*, *Prunus salicina*, *Psidium guajava*, *Pyrus* spp., *Ribes nigrum*, and *Syzygium samarangense*, which were obtained from supermarkets in Guangzhou, China. The fruit was washed with distilled water to remove dirt on the peel, and was given an airing at room temperature. The edible portion was separated by a kitchen knife. A precisely weighed amount (1 g) of the edible portion was mixed with 5 mL of distilled water, and ground into fine particles with a grinder, which mimicked the preparation of fruit juice. The sample was centrifuged at 5000× *g* for 10 min, and the supernatant was collected for animal study. The juice was stored at 4 °C for using within 1–2 days.

### 2.3. Detection of the Effects of 20 Fruits on Alcohol Metabolism in Mice

#### 2.3.1. Animal Study for Evaluation of Ethanol and Acetaldehyde Levels in Blood as Well as ADH and ALDH Activities in Liver

Seven-week-old male Kunming mice weighing 20–25 g were purchased from Laboratory Animal Center of Sun Yat-Sen University (Guangzhou, China). A total 126 Kunming mice were randomly divided into 21 groups with 6 mice in each group [13,14]. They were maintained in a room with a controlled temperature of 22 ± 0.5 °C, 40%–60% relative humidity, a 12 h light/dark cycle, and allowed free access to basal pellet diet and tap water. The study was carried out according to the Principles of Laboratory Animal Care and approved by the Institutional Animal Ethics Committee of Sun Yat-Sen University. All groups were given 52% (*v*/*v*) ethanol once at a dose of 4 g/kg body weight to induce acute alcohol intoxication. Subsequently, the control was given distilled water at a dose of 10 mL/kg body weight, while the fruit groups were treated once with different fruit juices at the same dose (equal to about 100 g fruit for a person of 60 kg). Two hours after the treatment, the mice were anesthetized. The blood was obtained and a piece of liver tissue was taken from each animal.

#### 2.3.2. Determination of Concentrations of Ethanol and Acetaldehyde in Blood

Blood samples (0.3 mL) were collected into 8 mL headspace vial which containing 1.2 mL 0.6 mol/L perchloric acid, 0.5 mL 10% trichloroacetic acid and 0.3 mL internal standard (160 mg/L tertiary butanol) for the determination of ethanol and acetaldehyde by using headspace-gas chromatography method according to the literature [15,16,17]. A Trace GC Ultra gas chromatograph (GC) (Finnigan, San Jose, CA, USA) was used. The vials containing samples were placed in an electric-heated thermostatic water bath at 70 °C for 30 min, and then 500 μL headspace gas was drawn by syringe and injected into GC column. The injection port temperature was 220 °C, and the FID detector temperature was 250 °C. The column oven temperature was kept at 40 °C for 5 min, and then programmed to increase from 40 °C to 240 °C at a rate of 40 °C/min. The flow rate of the carrier gas (nitrogen) was 0.4 mL/min (split ratio 25:1).

#### 2.3.3. Assay of ADH and ALDH Activities

Hepatic ADH and ALDH activities were evaluated according to Wu *et al.* [18,19]. Briefly, the liver tissues were weighed and homogenized at a 1:9 ratio (*w*/*v*) at 4 °C in 0.1 M Tris-HCl (pH 7.0). After centrifugation for 40 min at 13,000× *g* at 4 °C, the supernatants were collected for assay. The hepatic ADH activity was determined by monitoring the rate of NADH generation in the presence of ethanol in cuvettes maintained at 25 °C by a UV2550 spectrophotometer (Shimadzu, Kyoto, Japan) at 340 nm. The hepatic ALDH activity was determined by monitoring the rate of NADH generation in the presence of acetaldehyde at 30 °C.

### 2.4. Detection of the Effects of 20 Fruits on Acute Alcohol-Induced Liver Injury in Mice

#### 2.4.1. Animal Study for Evaluation of ALT and AST Activities in Serum, and SOD and MDA Levels in Liver

The 110 Kunming mice were randomly divided into 22 groups with five mice in each group [20,21]. The blank control group was given 0.2 mL of distilled water only. Other groups were given 52% (*v*/*v*) ethanol once at a dose of 6 g/kg body weight to induce acute alcohol intoxication [22,23,24]. Thirty minutes after alcohol administration, the model was given distilled water at a dose of 12 mL/kg body weight, while the fruit groups were treated once with different fruit juices at the same dose. The blood samples of mice were collected at 6 h after the last oral administration to determine the AST and ALT levels [25]. The liver was collected for the determinations of the activity of SOD and the content of MDA.

#### 2.4.2. Measurement of Hepatic Injury and Antioxidant Enzyme Activities

To estimate the ethanol-induced hepatotoxicity, ALT and AST activities in serum and the levels of MDA and SOD in liver were determined by the commercial detection kits according to the manufacturer’s instructions. 

### 2.5. Statistical Analysis

The results obtained were expressed as mean ± standard deviation (SD). Statistical significance was determined by one-way analysis of variance (ANOVA) followed by *post hoc* LSD test using SPSS 13.0 software (IBM, Armonk, NY, USA). The results were also analyzed by Pearson correlation. A value of *p* < 0.05 was considered statistically significant.

## 3. Results and Discussion

In the present study, the effects of 20 selected fruits on ethanol metabolism were studied to find out their potential health benefits and harmful impacts.

### 3.1. Effects of Fruit Juices on Concentrations of Ethanol and Acetaldehyde in Blood

The concentrations of blood ethanol and acetaldehyde are shown in Table 1. Generally, most of the fruit juices did not markedly affect the concentrations of blood ethanol and acetaldehyde. The concentrations of alcohol in blood ranged from 1334.19 ± 281.30 mg/L to 2361.98 ± 368.36 mg/L. *Citrus limon* (yellow), *Averrhoa carambola*, *Pyrus* spp., and *Syzygium samarangense* juices significantly (*p* < 0.05) decreased the concentrations of alcohol, while *Chaenomeles sinensis* juice increased the concentrations of alcohol in blood, which might increase the damage induced by alcohol. The concentrations of acetaldehyde ranged from 58.32 ± 3.51 mg/L to 83.59 ± 5.54 mg/L. Juices of *Citrus limon* (yellow), *Averrhoa carambola*, and *Syzygium samarangense* significantly (*p* < 0.05) increased the concentrations of acetaldehyde in blood.

Ethanol has a direct toxic effect on the hepatocyte and could induce severe liver perturbations of cholesterol and triglycerides metabolism [26]. Ethanol is also a neurotoxic agent that induces degeneration and damage to the brain, and alcohol drinking could impair the blood-brain barrier [27,28]. Since *Citrus limon* (yellow), *Averrhoa carambola*, *Pyrus* spp., and *Syzygium samarangense* juices could lower the concentration of ethanol in blood, consuming them might be beneficial when drinking alcohol. On the other hand, the results suggested that *Chaenomeles sinensis* should not be consumed with drinking alcohol because it could increase alcohol concentration in blood. The active components in these fruits and their mechanisms of action need to be studied further.

At high concentration, acetaldehyde has been shown to cause toxic effects, such as a rapid pulse, sweating, nausea, and vomiting, and cause morphologic damage to the pancreas [29]. Acetaldehyde may contribute to the pathological consequences of chronic alcohol intake for different forms of cancer in the digestive tract and the upper airways. Acetaldehyde seems to play a role in the etiology of liver cirrhosis and other pathological developments including brain damage, cardiomyopathy, pancreatitis, and fetal alcohol syndrome [30]. The results suggested that consuming *Citrus limon* (yellow), *Averrhoa carambola*, and *Syzygium samarangense* might increase the damage from drinking alcohol because they could increase the concentrations of acetaldehyde in blood.

### 3.2. Effects of Fruit Juices on Hepatic ADH and ALDH Activities

Most of fruits were studied for their effects on ADH and ALDH activities for the first time. As shown in Table 2, ADH and ALDH activities were decreased slightly by most of the fruit juices. *Prunus salicina* juice significantly (*p* < 0.05) decreased the activity of ADH by 15.62% ± 1.24%, while *Musa nana* juice significantly (*p* < 0.05) increased the activity of ADH by 16.50% ± 0.99%. In addition, juices of *Averrhoa carambola*, *Ribes nigrum*, *Lycopersivon esculentum*, *Chaenomeles sinensis*, and *Syzygium samarangense* significantly (*p* < 0.05) decreased the activity of ALDH by 61.95% ± 14.80%, 46.76% ± 19.30%, 46.14% ± 7.03%, 41.54% ± 9.88%, and 40.54% ± 9.56%, respectively.

About 90% of ethanol metabolism occurs in the liver, which is via the breakdown of ethanol to acetaldehyde and acetaldehyde to acetate by ADH and ALDH, respectively. *Musa nana* increased the activity of ADH. Perhaps this was because *Musa nana* has high content of potassium. In the literature, *Elaeagnus conferta* was reported to increase the activity of ADH, and also had a high ratio of K/Na [18]. *Mangifera indica* and *Durio zibethinus* juices didn’t significantly affect the activities of ADH and ALDH in this study, while it was reported in the literature that *Mangifera indica* fruit could increase the activities of ADH and ALDH and *Durio zibethinus* fruit extracts inhibited yeast ALDH activity *in vitro* [12,31]. These differences could be because of different extracts containing different components, and *in vitro* study and *in vivo* study sometimes showing different results. The results indicated that consuming some fruits such as *Averrhoa carambola*, *Ribes nigrum*, *Lycopersivon esculentum*, *Chaenomeles sinensis*, and *Syzygium samarangense* could increase the hangover symptoms because they could decrease the activity of ALDH after drinking alcohol.

*Citrus limon* (yellow) and *Pyrus* spp. decreased the concentrations of ethanol in blood. However, the activities of ADH and ALDH in these groups did not increase accordingly. The mechanism of decreasing the concentrations of ethanol in blood could be through other ways, such as the inhibition of ethanol absorption in the intestine. The results are in agreement with that reported by Kim and Park [32], where *Rhodiola sachalienesis* extract lowered the concentrations of ethanol in blood, while ADH and ALDH activities were not affected.

*Averrhoa carambola* and *Syzygium samarangense* juices increased the concentrations of acetaldehyde in blood. At the same time, the activities of ALDH were decreased by them (Table 2). It was assumed that *Averrhoa carambola* and *Syzygium samarangense* juices inhibited the activity of hepatic ALDH and, thus, slowed the clearance of acetaldehyde in blood and, hence, increased its toxicity. Therefore, treatment with *Averrhoa carambola* and *Syzygium samarangense* had similar effects with disulfiram, which is commonly used to treat alcohol use disorder [33].

**Figure 1:**
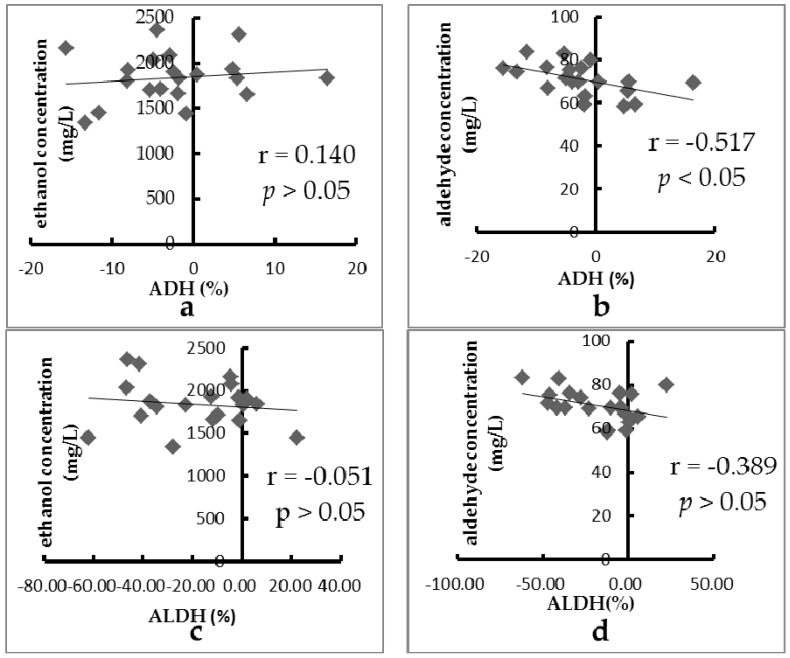
The relationship between the activity of ADH/ALDH and the concentration of ethanol/acetaldehyde in blood influenced by 20 fruits: (**a**) ADH and concentration of ethanol, (**b**) ADH and concentration of acetaldehyde, (**c**) ALDH and concentration of ethanol, and (**d**) ALDH and concentration of acetaldehyde.

The relationships between ADH activity and concentrations of ethanol and acetaldehyde in blood are shown in Figure 1a,b, respectively. No correlation (*r* = 0.140, *p* > 0.05) was obtained between the ADH activity and the concentration of ethanol in blood as influenced by the 20 fruits. A moderate negative correlation (*r* = −0.517, *p* < 0.05) between the ADH activity and the concentration of acetaldehyde in blood was also obtained in this study. In addition, the relationship between ALDH activity and concentrations of ethanol and acetaldehyde in blood are shown in Figure 1c,d, respectively. No correlation (*r* = −0.051, *p* > 0.05 or *r* = −0.389, *p* > 0.05) was found between the ALDH activity and the concentration of ethanol or acetaldehyde in blood as influenced by the 20 fruits. The results suggested that these fruits that influenced the concentrations of ethanol and acetaldehyde in blood were not by means of changing the activities of ADH and ALDH, but through other ways, such as non-enzymatic ways, which could change the absorption of ethanol in the stomach and intestine or mediate the excretion of ethanol through breath and urine.

### 3.3. Effects of Fruit Juices on Levels of ALT and AST in Serum

ALT and AST are the two important aminopherases, and they mostly reside in endochylema and mitochondrion. After heavy drinking, alcohol and its main metabolite, acetaldehyde, may have a direct toxic effect on the hepatocyte. They could raise the permeability of the cell membrane, leading to the emission of aminopherase, which increased the contents of the two enzymes in serum. Aminopherase content in blood is one of the most direct and sensitive indexes reflecting the damage of hepatic cell [34].

The activities of ALT and AST are shown in Table 3. *Citrus limon* (yellow) offered significant protection against acute alcohol-intoxicated mice by attenuating ALT and AST elevation induced by alcohol (*p* < 0.05). *Cucumis melo* significantly (*p* < 0.05) decreased the level of ALT, while *Hylocereus undulates* increased it. Additionally, *Averrhoa carambola* and *Musa nana* significantly (*p* < 0.05) decreased the level of AST, while *Ribes nigrum* showed the opposite result.

Reduction in the levels of AST and ALT to the normal value indicated the stabilization of plasma membrane and the repair of hepatic tissue. It was reported in the literature that *Citrus limon* extracts showed protective effects on carbon tetrachloride (CCl_4_)-induced liver damage in rats and CCl_4_-exposed HepG2 cell lines [35], which was similar to the results from this study. The high contents of hesperidin, luteolin, kaempferol, which are important flavonoids, were found in lemon [36]. According to the literature, flavonoids could be potent preventive agents against acute alcohol-induced liver injury [25,37,38]. Therefore, hesperidin, luteolin, and kaempferol could be the active components in lemon.

ALT mainly presents in liver, whereas AST can be found in liver, skeletal muscle, cardiac muscle, pancreas, kidney, brain, and lung. ALT is more specific for hepatic damage with respect to AST. Therefore, *Cucumis melo* could be more efficient than *Averrhoa carambola* and *Musa nana* for protecting the liver. However, *Ribes nigrum* and *Hylocereus undulates* could increase the liver damage induced by alcohol because they increased AST or ALT level in blood. Therefore, it is not advised to consume *Ribes nigrum* and *Hylocereus undulates* after drinking alcohol*.*

**Figure 2:**
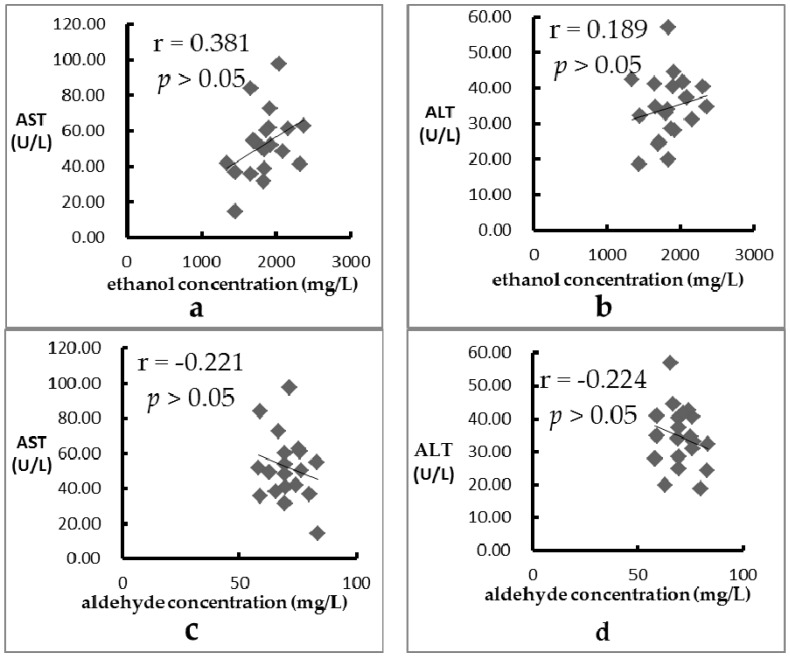
The relationship between the concentration of ethanol/acetaldehyde in blood and the level of AST/ALT influenced by 20 fruits: (**a**) concentration of ethanol and level of AST, (**b**) concentration of ethanol and level of ALT; (**c**) concentration of acetaldehyde and level of AST; and (**d**) concentration of acetaldehyde and level of ALT.

The relationship between the concentration of ethanol in blood and the levels of AST and ALT influenced by 20 fruits are shown in Figure 2a,b, respectively. In addition, the relationship between the concentration of acetaldehyde in blood and the levels of AST and ALT are shown in Figure 2c,d, respectively. No correlation was found between ethanol concentration and the level of AST (*r* = 0.381, *p* > 0.05) as well as between ethanol concentration and the level of ALT (*r* = 0.189, *p* > 0.05). In addition, no correlations were found between acetaldehyde concentration and the levels of AST and ALT (*r* = −0.221, *p* > 0.05 and *r* = −0.224, *p* > 0.05, respectively). The results suggested that effects of these fruits on alcohol induced liver damage were not by regulating alcohol metabolism, but through other ways, such as protecting against oxidative stress.

### 3.4. Effects of Fruit Juices on MDA Level and SOD Activity in Liver

In normal conditions, liver possess enzymatic (SOD, CAT, glutathion peroxidase) and nonenzymatic antioxidants (glutathione), as a powerful antioxidant defense system [39]. Alcohol metabolism could increase the production of reactive oxygen species (ROS), and enhance peroxidation of lipids, proteins, and DNA, thus increasing the hepatocellular damage. The impairment of liver SOD, CAT, glutathion peroxidase activities, and glutathione content were reported in alcoholics and experimental animals [40,41,42]. Previous studies showed that ethanol-induced liver damage might be mediated by oxidative stress and nutritional deficiency [43]. The importance of oxidative stress in liver damage is widely accepted [44,45]. The generation of lipid peroxidation by free radicals has been suggested to be a mechanism of ethanol-induced hepatotoxicity. MDA is one of the main end products of lipid peroxidation, and could reflect the preoxidative degree of lipid *in vivo*. Therefore, changes in the content of MDA could indicate the damage of cells indirectly. In consistent with previous report [46], the results of this study also showed that acute ethanol administration significantly increased the MDA level in liver (Table 4). In addition, the SOD level in liver decreased only slightly (*p* > 0.05) in alcohol-treated mice compared with the control group in the present study, which was similar to the previous report [43].

As shown in Table 4, the levels of hepatic MDA significantly (*p* < 0.05) increased by *Citrus limon* (yellow) and *Passionfora edulis*. The elevation of liver MDA concentration induced by alcohol was lowered significantly (*p* < 0.05) by treated with juices of *Averrhoa carambola*, *Garcinia mangostana*, *Citrullus lanatus*, *Prunus salicina*, and *Durio zibethinus*. Hepatic SOD levels were decreased significantly (*p* < 0.05) by *Prunus salicina* and *Musa nana*, but *Syzygium samarangense* significantly (*p* < 0.05) increased the SOD levels in liver.

MDA is characterized by cross-linking cellular macromolecules including proteins and DNA, and induces widespread cellular damage. Consuming *Passionfora edulis* after drinking alcohol could increase lipid peroxidation in the liver. The level of hepatic MDA was also increased by *Citrus limon* (yellow), which could result from the increased acetaldehyde concentration according to Table 1. In the literature, *Averrhoa carambola and Prunus salicina* fruit extracts treatment brought out a significant reduction in lipid peroxidation compared to the carcinogen-treated control [47,48], which were consistent with the results obtained in this study. Kaempferol, myricitin, campherol, and cinnamic acid were found to be main phenolic compounds in *Durio zibethinus.* Meanwhile, kaempferol, quercetin-3-glucoside, and caffeic acid were found in *Prunus salicina*, and chlorogenic acid and luteolin were found in *Citrullus lanatus.* In addition, quercetin was main phenolic compound in *Garcinia mangostana* [36]. Since many flavonoids, including caffeic acid and quercetin, were found to have inhibitory activity on lipid peroxidation [49,50], they could contribute to the effects of decreasing MDA content by these fruits.

*Prunus salicina* and *Musa nana* decreased hepatic SOD levels. Superoxide anion produced during oxidative stress could be converted to hydrogen peroxide by SOD, which is a metalloenzyme. Decreased hepatic SOD levels might affect the convert of superoxide anion, which might lead to the accumulation of these highly reactive free radicals, resulting in deleterious effects including loss of cell membrane integrity and function [51]. Treatment with juice of *Syzygium samarangense* exhibited protection against alcohol-induced hepatic SOD depletion, as evidenced by reversing to approximate the normal level. The effect could be related with the decreased ethanol level as shown in Table 1. *Syzygium samarangense* is the ultimate sources of anthocyanin, carotene, and vitamin C [52], which have important antioxidant activities. Meanwhile, anthocyanins could be detected in human serum after consuming blueberries [53]. Anthocyanin, carotene, and vitamin C could contribute to the increase of SOD levels treated with *Syzygium samarangense*.

## 4. Conclusions

The effects of 20 selected fruits on ethanol metabolism and acute alcohol-induced liver injury in mice were evaluated. Generally, the effects of 20 fruits on ethanol metabolism and alcohol-induced liver injury were very different. The results suggested that some fruits should not be consumed accompanied by drinking alcohol, such as *Chaenomeles sinensis*, *Hylocereus undulates*, *Ribes nigrum*, and *Passionfora edulis*, because they could aggravate liver damage induced by alcohol*.* However, several fruits, such as *Pyrus* spp. and *Cucumis melo*, could be potential dietary supplements for the prevention of harm from alcohol consumption. In addition, some fruits, such as *Averrhoa carambola* and *Syzygium samarangense*, could be developed as functional food or drug to treat alcohol use disorder because they could decrease the activity of ALDH and increase the concentration of acetaldehyde in blood. The results could give advice on the choice of fruit after drinking alcohol for the public.

## Figures and Tables

**Table 1:** The concentrations of ethanol and acetaldehyde in blood in different groups.

Group	Alcohol Concentration (mg/L)	Acetaldehyde Concentration (mg/L)
Control	1901.17 ± 296.83	69.17 ± 5.33
*Actinidia chinensis*	1653.83 ± 136.57	58.99 ± 5.12
*Averrhoa carambola*	1450.91 ± 264.36 *	83.59 ± 5.54 *
*Chaenomeles sinensis*	2312.84 ± 366.21 *	69.73 ± 5.10
*Citrullus lanatus*	1917.44 ± 206.95	66.81 ± 7.59
*Citrus limon* (green)	1708.20 ± 273.53	69.51 ± 8.67
*Citrus limon* (yellow)	1444.19 ± 293.45 *	79.94 ± 7.27 *
*Citrus sinensis*	1661.93 ± 354.28	59.15 ± 4.66
*Cucumis melo*	1835.23 ± 174.74	62.90 ± 4.40
*Durio zibethinus*	2080.69 ± 156.26	69.63 ± 10.66
*Garcinia mangostana*	1870.44 ± 239.21	69.52 ± 8.58
*Hylocereus undulatus*	1836.58 ± 92.34	65.51 ± 6.20
*Lycopersivon esculentum*	2361.98 ± 368.36	75.34 ± 10.31
*Mangifera indica*	1928.60 ± 115.76	58.32 ± 3.51
*Musa nana*	1826.99 ± 260.28	69.31 ± 3.34
*Passionfora edulis*	1804.23 ± 335.73	76.44 ± 20.31
*Prunus salicina*	2161.10 ± 363.66	76.19 ± 5.55
*Psidium guajava*	1900.77 ± 496.96	75.86 ± 12.72
*Pyrus* spp.	1334.19 ± 281.30 *	74.22 ± 7.86
*Ribes nigrum*	2037.17 ± 300.63	71.59 ± 4.02
*Syzygium samarangense*	1699.99 ± 71.89 *	82.93 ± 5.31 *

* Means the levels of the parameters in the group were significantly different (*p* < 0.05) from that of the control.

**Table 2:** Effects of the 20 fruit juices on ADH and ALDH activities.

Group	ADH (%)	ALDH (%)
control	−	−
*Actinidia chinensis*	6.65 ± 0.13	−1.01 ± 0.50
*Averrhoa carambola*	−11.57 ± 0.28	−61.95 ± 14.80 *
*Chaenomeles sinensis*	5.63 ± 0.34	−41.54 ± 9.88 *
*Citrullus lanatus*	−8.00 ± 1.08	−1.45 ± 0.65
*Citrus limon* (green)	−3.94 ± 0.92	−9.89 ± 2.62
*Citrus limon* (yellow)	−0.89 ± 0.03	22.24 ± 7.91
*Citrus sinensis*	−1.93 ± 0.18	−11.81 ± 2.32
*Cucumis melo*	−1.79 ± 0.11	0.53 ± 0.17
*Durio zibethinus*	−2.84 ± 0.26	−4.42 ± 0.85
*Garcinia mangostana*	0.46 ± 0.06	−36.97 ± 16.79
*Hylocereus undulatus*	5.37 ± 0.53	6.09 ± 1.20
*Lycopersivon esculentum*	−4.39 ± 0.79	−46.14 ± 7.03 *
*Mangifera indica*	4.85 ± 0.13	−12.33 ± 3.23
*Musa nana*	16.50 ± 0.99 *	−22.75 ± 7.17
*Passionfora edulis*	−8.16 ± 0.81	−34.30 ± 12.92
*Prunus salicina*	−15.62 ± 1.24 *	−4.63 ± 1.05
*Psidium guajava*	−2.46 ± 0.18	1.98 ± 0.44
*Pyrus* spp.	−13.27 ± 1.30	−27.71 ± 7.72
*Ribes nigrum*	−4.94 ± 0.96	−46.76 ± 19.30 *
*Syzygium samarangense*	−5.38 ± 1.02	−40.54 ± 9.56 *

* Means the levels of the parameters in the group were significantly different (*p* < 0.05) from that of the control.

**Table 3:** Effect of fruit juices on serum levels of ALT and AST in alcohol-treated mice.

Group	AST (U/L)	ALT (U/L)
Blank control	42.65 ± 10.21	32.38 ± 16.72
Model	55.28 ± 11.23 **	35.88 ± 11.04 **
*Actinidia chinensis*	83.90 ± 54.89	41.02 ± 10.76
*Averrhoa carambola*	14.37 ± 14.35 *	32.22 ± 9.05
*Chaenomeles sinensis*	40.93 ± 10.21	40.40 ± 10.16
*Citrullus lanatus*	72.42 ± 27.10	44.40 ± 14.97
*Citrus limon* (green)	54.00 ± 12.19	24.84 ± 7.02
*Citrus limon* (yellow)	36.42 ± 8.25 *	18.75 ± 5.61 *
*Citrus sinensis*	35.37 ± 13.14	34.76 ± 12.69
*Cucumis melo*	49.19 ± 2.49	19.91 ± 3.70 *
*Durio zibethinus*	48.34 ± 13.18	37.38 ± 8.48
*Garcinia mangostana*	59.82 ± 10.78	28.50 ± 13.08
*Hylocereus undulatus*	38.37 ± 11.39	56.98 ± 14.59 *
*Lycopersivon esculentum*	62.82 ± 6.44	34.67 ± 1.70
*Mangifera indica*	51.78 ± 16.17	28.01 ± 4.77
*Musa nana*	31.35 ± 3.70 *	33.99 ± 12.91
*Passionfora edulis*	50.38 ± 13.12	33.02 ± 8.53
*Prunus salicina*	61.30 ± 37.38	31.07 ± 16.42
*Psidium guajava*	61.36 ± 7.75	40.48 ± 14.90
*Pyrus* spp.	41.80 ± 7.74	42.45 ± 15.09
*Ribes nigrum*	97.38 ± 36.33 *	41.68 ± 12.12
*Syzygium samarangense*	54.65 ± 21.65	24.24 ± 4.66

* Means the levels of the parameters in the group were significantly different (*p* < 0.05) from that of the model; ** Means the levels of the parameters in the model were significantly different (*p* < 0.05) from that of the blank control.

**Table 4:** Effects of fruit juices on levels of MDA and SOD in alcohol-treated mice.

Group	MDA (nmol/mg·prot)	SOD (U/mL)
Blank control	0.75 ± 0.08	59.49 ± 3.60
Model	0.86 ± 0.16 **	57.83 ± 7.62
*Actinidia chinensis*	0.83 ± 0.27	59.62 ± 9.99
*Averrhoa carambola*	0.65 ± 0.07 *	59.00 ± 3.02
*Chaenomeles sinensis*	1.14 ± 0.45	56.93 ± 6.17
*Citrullus lanatus*	0.60 ± 0.07 *	60.57 ± 3.07
*Citrus limon* (green)	0.76 ± 0.19	61.90 ± 6.36
*Citrus limon* (yellow)	1.19 ± 0.11 *	54.62 ± 4.29
*Citrus sinensis*	0.78 ± 0.08	52.33 ± 3.37
*Cucumis melo*	0.71 ± 0.17	53.71 ± 2.81
*Durio zibethinus*	0.59 ± 0.07 *	63.60 ± 3.23
*Garcinia mangostana*	0.62 ± 0.06 *	56.98 ± 3.96
*Hylocereus undulatus*	1.00 ± 0.25	51.01 ± 7.61
*Lycopersivon esculentum*	0.77 ± 0.02	52.11 ± 6.99
*Mangifera indica*	0.81 ± 0.16	60.22 ± 4.96
*Musa nana*	0.85 ± 0.08	48.32 ± 2.74 *
*Passionfora edulis*	1.33 ± 0.21 *	54.08 ± 6.43
*Prunus salicina*	0.55 ± 0.13 *	51.56 ± 3.28 *
*Psidium guajava*	1.00 ± 0.15	64.98 ± 1.05
*Pyrus* spp.	0.61 ± 0.12	63.65 ± 5.63
*Ribes nigrum*	1.04 ± 0.36	55.25 ± 3.35
*Syzygium samarangense*	1.08 ± 0.18	68.28 ± 4.26 *

* Means the levels of the parameters in the group were significantly different (*p* < 0.05) from that of the model; ** Means the levels of the parameters in the model were significantly different (*p* < 0.05) from that of the blank control.

## Abbreviations

The following abbreviations are used in this manuscript:

AST	aspartate transaminase
ALT	alanine transferase
ALDH	acetaldehyde dehydrogenase
ADH	alcohol dehydrogenase
ROS	reactive oxygen species
NAD	β-nicotinamide adenine dinucleotide
MDA	malondialdehyde
SOD	superoxide dismutase
GC	Gas Chromatograph
CCl_4_	carbon tetrachloride

## References

[1] Sung C., Kim S., Oh C., Yang S., Han B., Mo E. (2012). Taraxerone enhances alcohol oxidation via increases of alcohol dehyderogenase (ADH) and acetaldehyde dehydrogenase (ALDH) activities and gene expressions. Food Chem. Toxicol..

[2] Chiba T., Phillips S.F. (2000). Alcohol-related diarrhea. Addict. Biol..

[3] Li S., Gan L.Q., Li S.K., Zheng J.C., Xu D.P., Li H.B. (2014). Effects of herbal infusions, tea and carbonated beverages on alcohol dehydrogenase and aldehyde dehydrogenase activity. Food Funct..

[4] Jung Y.S., Kim S.J., Kwon D.Y., Ahn C.W., Kim Y.S., Choi D.W., Kim Y.C. (2013). Alleviation of alcoholic liver injury by betaine involves an enhancement of antioxidant defense via regulation of sulfur amino acid metabolism. Food Chem. Toxicol..

[5] Bourogaa E., Nciri R., Mezghani-Jarraya R., Racaud-Sultan C., Damak M., El Feki A. (2013). Antioxidant activity and hepatoprotective potential of *Hammada scoparia* against ethanol-induced liver injury in rats. J. Physiol. Biochem..

[6] Kim B.Y., Cui Z.G., Lee S.R., Kim S.J., Kang H.K., Lee Y.K., Park D.B. (2009). Effects of *Asparagus officinalis* extracts on liver cell toxicity and ethanol metabolism. J. Food Sci..

[7] Arteel G.E. (2003). Oxidants and antioxidants in alcohol-induced liver disease. Gastroenterology.

[8] Grasselli E., Compalati A.D., Voci A., Vecchione G., Ragazzoni M., Gallo G., Borro P., Sumberaz A., Testino G., Vergani L. (2014). Altered oxidative stress/antioxidant status in blood of alcoholic subjects is associated with alcoholic liver disease. Drug Alcohol Depen..

[9] Fu L., Xu B.T., Xu X.R., Qin X.S., Gan R.Y., Li H.B. (2010). Antioxidant capacities and total phenolic contents of 56 wild fruits from south China. Molecules.

[10] Deng G.F., Shen C., Xu X.R., Kuang R.D., Guo Y.J., Zeng L.S., Gao L.L., Lin X., Xie J.F., Xia E.Q. (2012). Potential of fruit wastes as natural resources of bioactive compounds. Int. J. Mol. Sci..

[11] Reddy V.D., Padmavathi P., Varadacharyulu N.C. (2009). *Emblica officinalis* protects against alcohol-induced liver mitochondrial dysfunction in rats. J. Med. Food.

[12] Maninang J.S., Lizada M.C.C., Gemma H. (2009). Inhibition of aldehyde dehydrogenase enzyme by durian (*Durio zibethinus* Murray) fruit extract. Food Chem..

[13] Liu Y., Yang X., Jing Y.Y., Zhang S.S., Zong C., Jiang J.H., Sun K., Li R., Gao L., Zhao X. (2015). Contribution and mobilization of mesenchymal stem cells in a mouse model of carbon tetrachloride-induced liver fibrosis. Sci. Rep..

[14] Pisonero-Vaquero S., Martinez-Ferreras A., Victoria Garcia-Mediavilla M., Martinez-Florez S., Fernandez A., Benet M., Olcoz L.J., Jover R., Gonzalez-Gallego J., Sanchez-Campos S. (2015). Quercetin ameliorates dysregulation of lipid metabolism genes via the PI3K/AKT pathway in a diet-induced mouse model of nonalcoholic fatty liver disease. Mol. Nutr. Food Res..

[15] Lee H., Isse T., Kawamoto T., Woo H., Kim A.K., Park J.Y., Yang M. (2012). Effects and action mechanisms of Korean pear (*Pyrus pyrifolia*
*cv.* Shingo) on alcohol detoxification. Phytother. Res..

[16] Isse T., Matsuno K., Oyama T., Kitagawa K., Kawamoto T. (2005). Aldehyde dehydrogenase 2 gene targeting mouse lacking enzyme activity shows high acetaldehyde level in blood, brain, and liver after ethanol gavages. Alcohol Clin. Exp. Res..

[17] Zhang X.Q., Xu D.P., Li A.N., Zhang Y.J., Wang F., Zheng J., Li S., Li H.B. (2014). Selection and evaluation of biochemical parameters for antialcohol and hepatoprotective effects of foods and traditional medicines. Int. J. Modern Biol. Med..

[18] Wu C., Dai R., Bai J., Chen Y., Yu Y., Meng W., Deng Y. (2011). Effect of *Elaeagnus Conferta* Roxb (Elaeagnaceae) dry fruit on the activities of hepatic alcohol dehydrogenase and aldehyde dehydrogenase in mice. Trop. J. Pharm. Res..

[19] Lindahl R., Evces S. (1987). Changes in aldehyde dehydrogenase activity during diethylnitrosamine-initiated rat hepatocarcinogenesis. Carcinogenesis.

[20] Coombes J.D., Swiderska-Syn M., Dolle L., Reid D., Eksteen B., Claridge L., Briones-Orta M.A., Shetty S., Oo Y.H., Riva A. (2015). Osteopontin neutralisation abrogates the liver progenitor cell response and fibrogenesis in mice. Gut.

[21] Park M., Kim Y.H., Woo S.Y., Lee H.J., Yu Y., Kim H.S., Park Y.S., Jo I., Park J.W., Jung S.C. (2015). Tonsil-derived mesenchymal stem cells ameliorate CCl_4_-induced liver fibrosis in mice via autophagyactivation. Sci. Rep..

[22] Wang Z.J., Wu X.L., Zhang Y.S., Zhou L., Li L.N., Yu Y.L., Wang L.Y. (2012). Discrepant roles of CpG ODN on acute alcohol-induced liver injury in mice. Int. Immunopharmacol..

[23] Zhao J., Chen H., Li Y. (2008). Protective effect of bicyclol on acute alcohol-induced liver injury in mice. Eur. J. Pharmacol..

[24] Yang R.K., Han X.N., Delude R.L., Fink M.P. (2003). Ethyl pyruvate ameliorates acute alcohol-induced liver injury and inflammation in mice. J. Lab. Clin. Med..

[25] Wang J.M., Zhang Y.Y., Zhang Y.S., Cui Y., Liu J., Zhang B.F. (2012). Protective effect of *Lysimachia christinae* against acute alcohol-induced liver injury in mice. Biosci Trends.

[26] Alimi H., Hfaeidh N., Mbarki S., Bouoni Z., Sakly M., Ben Rouma K. (2012). Evaluation of *Opuntia ficus* Indica F. inermis fruit juice hepatoprotective effect upon ethanol toxicity in rats. Gen. Physiol. Biophys..

[27] Costa P.A., Poli J.H.Z., Sperotto N.D.M., Moura D.J., Saffi J., Nin M.S., Barros H.M.T. (2015). Brain DNA damage and behavioral changes after repeated intermittent acute ethanol withdrawal by young rats. Psychopharmacology.

[28] Haorah J., Knipe B., Gorantla S., Zheng J., Persidsky Y. (2007). Alcohol-induced blood-brain barrier dysfunction is mediated via inositol 1,4,5-triphosphate receptor (IP3R)-gated intracellular calcium release. J. Neurochem..

[29] Jelski W., Kutylowska E., Laniewska-Dunaj M., Orywal K., Laszewicz W., Szmitkowski M. (2011). Alcohol dehydrogenase (ADH) isoenzymes and aldehyde dehydrogenase (ALDH) activity in the sera of patients with acute and chronic pancreatitis. Exp. Mol. Pathol..

[30] Eriksson C. (2001). The role of acetaldehyde in the actions of alcohol (update 2000). Alcohol. Clin. Exp. Res..

[31] Kim S.H., Cho S.K., Min T.S., Kim Y., Yang S.O., Kim H.S., Hyun S.H., Kim H., Kim Y.S., Choi H.K. (2011). Ameliorating effects of mango (*Mangifera indica* L.) fruit on plasma ethanol level in a mouse model assessed with H-1-NMR based metabolic profiling. J. Clin. Biochem. Nutr..

[32] Kim M.H., Park C.K. (1997). Inhibition of ethanol absorption by *Rhodiola sachalinensis* in rats. Arch. Pharm. Res..

[33] Kim A.K., Souza-Formigoni M. (2010). Disulfiram impairs the development of behavioural sensitization to the stimulant effect of ethanol. Behav. Brain Res..

[34] Dolganiuc A., Szabo G. (2009). *In vitro* and *in vivo* models of acute alcohol exposure. World J. Gastroenterol..

[35] Bhavar S.K., Joshi P., Shah M.B., Santani D.D. (2007). Investigation into hepatoprotective activity of *Citrus limon*. Pharm. Biol..

[36] Fu L., Xu B.T., Xu X.R., Gan R.Y., Zhang Y., Xia E.Q., Li H.B. (2011). Antioxidant capacities and total phenolic contents of 62 fruits. Food Chem..

[37] Wang H., Feng F., Zhuang B.Y., Sun Y. (2009). Evaluation of hepatoprotective effect of Zhi-Zi-Da-Huang decoction and its two fractions against acute alcohol-induced liver injury in rats. J. Ethnopharmacol..

[38] Wu S.K., Zhang N., Shen X.R., Mei W.W., He Y., Ge W.H. (2015). Preparation of total flavonoids from loquat flower and its protective effect on acute alcohol-induced liver injury in mice. J. Food Drug Anal..

[39] Jurczuk M., Brzoska M.M., Moniuszko-Jakoniuk J., Galazyn-Sidorczuk M., Kulikowska-Karpinska E. (2004). Antioxidant enzymes activity and lipid peroxidation in liver and kidney of rats exposed to cadmium and ethanol. Food Chem. Toxicol..

[40] Wang M., Zhu P., Jiang C., Ma L., Zhang Z., Zeng X. (2012). Preliminary characterization, antioxidant activity *in vitro* and hepatoprotective effect on acute alcohol-induced liver injury in mice of polysaccharides from the peduncles of *Hovenia dulcis*. Food Chem. Toxicol..

[41] Yao P., Li K., Jin Y., Song F.F., Zhou S.L., Sun X.F., Nussler A.K., Liu L.G. (2006). Oxidative damage after chronic ethanol intake in rat tissues: Prophylaxis of *Ginkgo biloba* extract. Food Chem..

[42] Husain K., Scott B.R., Reddy S.K., Somani S.A. (2001). Chronic ethanol and nicotine interaction on rat tissue antioxidant defense system. Alcohol.

[43] Cheng N., Du B., Wang Y., Gao H., Cao W., Zheng J., Feng F. (2014). Antioxidant properties of jujube honey and its protective effects against chronic alcohol-induced liver damage in mice. Food Funct..

[44] Wang F., Li Y., Zhang Y.J., Zhou Y., Li S., Li H.B. (2016). Natural products for the prevention and treatment of hangover and alcohol use disorder. Molecules.

[45] Li S., Tan H.Y., Wang N., Zhang Z.J., Lao L.X., Wong C.W., Feng Y.B. (2015). The role of oxidative stress and antioxidants in liver diseases. Int. J. Mol. Sci..

[46] Zhao M., Du Y.Q., Yuan L., Wang N.N. (2010). Protective effect of puerarin on acute alcoholic liver injury. Am. J. Chin. Med..

[47] Singh R., Sharma J., Goyal P.K. (2014). Prophylactic role of *Averrhoa carambola* (star fruit) extract against chemically induced hepatocellular carcinoma in Swiss albino mice. Adv. Pharmacol. Sci..

[48] Kim H.J., Yu M.H., Lee I.S. (2008). Inhibitory effects of methanol extract of plum (*Prunus salicina* L., cv. “Soldam”) fruits against benzo(α)pyrene-induced toxicity in mice. Food Chem. Toxicol..

[49] Mitra I., Saha A., Roy K. (2012). *In silico* development, validation and comparison of predictive QSAR models for lipid peroxidation inhibitory activity of cinnamic acid and caffeic acid derivatives using multiple chemometric and cheminformatics tools. J. Mol. Model.

[50] Surapaneni K.M., Jainu M. (2014). Comparative effect of pioglitazone, quercetin and hydroxy citric acid on the status of lipid peroxidation and antioxidants in experimental non-alcoholic steatohepatitis. J. Physiol. Pharmacol..

[51] Reddy A.C., Lokesh B.R. (1992). Studies on spice principles as antioxidants in the inhibition of lipid peroxidation of rat liver microsomes. Mol. Cell Biochem..

[52] Khandaker M.M., Sarwar J.M., Mat N., Boyce A.N. (2015). Bioactive constituents, antioxidant and antimicrobial activities of three cultivars of wax apple (*Syzygium samarangense* L.) fruits. Res. J. Biotechnol..

[53] Zhang Y.J., Gan R.Y., Li S., Zhou Y., Li A.N., Xu D.P., Li H.B. (2015). Antioxidant phytochemicals for the prevention and treatment of chronic diseases. Molecules.

